# Assessing germline *TP53* mutations in cancer patients: insights into Li-Fraumeni syndrome and genetic testing guidelines

**DOI:** 10.1186/s13053-025-00307-w

**Published:** 2025-02-17

**Authors:** Anastasiia Danishevich, Daria Fedorova, Natalia Bodunova, Maria Makarova, Maria Byakhova, Anna Semenova, Vsevolod Galkin, Maria Litvinova, Sergey Nikolaev, Irina Efimova, Pavel Osinin, Tatyana Lisitsa, Anastasiya Khakhina, German Shipulin, Tatiana Nasedkina, Syuykum Shumilova, Oleg Gusev, Airat Bilyalov, Elena Shagimardanova, Leyla Shigapova, Marina Nemtsova, Olesya Sagaydak, Mary Woroncow, Saida Gadzhieva, Igor Khatkov

**Affiliations:** 1 SBHI Moscow Clinical Scientific Center Named After Loginov of Moscow Healthcare Department, Moscow, 111123 Russia; 2 Evogen LLC, Moscow, 115191 Russia; 3 Russian Scientific Center of Roentgenoradiology of the Ministry of Health of the Russian Federation, Moscow, 117997 Russia; 4 City Clinical Oncological Hospital No. 1 of Moscow Healthcare Department, Moscow, 117152 Russia; 5 Federal State Autonomous Educational Institution of Higher Education I.M. Sechenov First Moscow State Medical University of the Ministry of Health of Russian Federation (Sechenov University), Moscow, 119991 Russia; 6 Medical Genetic Research Center Named After Academician N.P. Bochkov, Moscow, 115522 Russia; 7 FSBI ”Centre for Strategic Planning and Management of Biomedical Health Risks” of the Federal Medical and Biological Agency, Moscow, 119435 Russia; 8 FSBI ”National Medical Research Center of Oncology Named After N.N. Blokhin” of the Ministry of Health of the Russian Federation, Moscow, 115522 Russia; 9 Engelhardt Institute of Molecular Biology of the Russian Academy of Sciences, Moscow, 119991 Russia; 10 Life Improvement By Future Technologies (LIFT) Center, Skolkovo, Moscow, 143025 Russia; 11 Kazan Federal University, Kazan, 420008 Russia; 12 National Medical Research Center of Endocrinology, Moscow, 117292 Russia; 13 Lomonosov Moscow State University, Moscow, 119991 Russia; 14 Moscow Healthcare Department, Moscow, 127006 Russia

**Keywords:** *TP53*, NGS, Li-Fraumeni syndrome, Germline mutations

## Abstract

**Background:**

Germline *TP53* gene variants are intricately linked to Li-Fraumeni syndrome, a rare and aggressive hereditary cancer syndrome. This study investigated the frequency and spectrum of *TP53* pathogenic variants associated with Li-Fraumeni syndrome in a large cohort of mainly breast cancer patients from Russia.

**Methods:**

The study analyzed 3,455 genomic DNA samples from cancer patients using next-generation sequencing panels and whole-genome sequencing. Clinically significant *TP53* variants were identified and validated using Sanger sequencing. The clinical and family history characteristics of patients with *TP53* variants were analyzed.

**Results:**

The analysis identified 13 (0.4%) individuals with clinically significant germline *TP53* variants, all of whom were females with either unilateral breast cancer or breast cancer as part of multiple primary malignant neoplasms. The average age of breast cancer manifestation was 39.9 years, with a median of 36 years. Only 38.5% of the *TP53* mutation carriers met the modified Chompret criteria for *TP53* testing.

**Conclusions:**

The findings underscore the necessity of thorough phenotype and family history analysis in genetic counseling to effectively diagnose LFS, and emphasize the importance of identifying *TP53* variant carriers for developing treatment strategies, prognosis, and monitoring, as well as for identifying high-risk family members. The study also highlights that the current guidelines fail to identify over half of the *TP53* mutation carriers, suggesting the need for a more comprehensive approach to genetic testing in suspected hereditary cancer cases.

## Background

Hereditary malignant neoplasms (MN) are observed across virtually all primary tu-mor sites, constituting an average of 10% of all initially detected tumors. The heightened predisposition to oncological diseases stems from the presence of germline mutations in oncogenes and tumor suppressor genes. These mutations significantly contribute to the development of hereditary cancer syndromes (HCS). HCS often manifest with a specific spectrum of MN, wherein the risk of developing cancer in specific locations varies from moderate to high, depending upon the affected gene and the type of structural aberration, thereby determining the degree of cancer awareness in each particular case.

One of the most significant HCS is the *TP53*-associated cancer syndrome, also known as Li-Fraumeni Syndrome (LFS). LFS was initially described by Joseph Fraumeni and Frederick Li in 1969, following a retrospective analysis of a cohort of children with rhabdomyosarcoma. Subsequently, an autosomal dominant inheritance pattern was identified for LFS. The exact prevalence of the disease remains undetermined to date. LFS is characterized by early onset and a wide spectrum of tumors with the most common including soft tissue sarcomas (26.4%), central nervous system (CNS) tumors (13.1%), soft tissue sarcomas (11.6%), osteosarcomas (9.1%), adrenocortical carcinoma (5.2%), hematologic malignancies (4.7%), colorectal cancer (3.6%), lung cancer (3.6%), gastric cancer (3.1%), alongside MN at other sites (19.4%) [1]. The cumulative risk of developing at least one neoplasm reaches 40–50% by the age of 30 and 80–100% – by the age of 70 [1, 2]. Germline mutations in the *TP53* gene serve as the initiating event in the development of LFS, defining the initial stages of carcinogenesis. In 70–80% of cases with clinical manifestations of LFS, germline mutations in the *TP53* gene are detected [3], while estimates suggest that the contribution of de novo mutation variants ranges from 7 to 20% out of identified cases [4]. Somatic mutations in *TP53* are also considered the most prevalent alteration in the formation of many types of MN and can be detected in 50% of tumors [5].

**Fig. 1:**
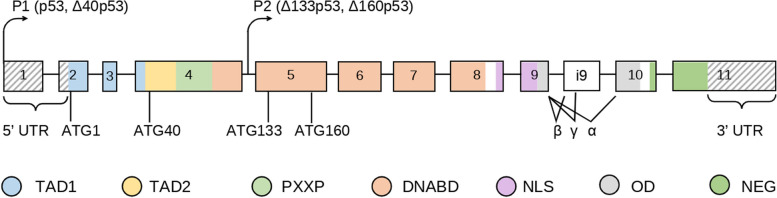
Structure of the *TP53* gene and different isoforms of the p53 protein. The N-terminal domains TAD1 and TAD2 (transactivation domain) are required for the activation of functionally related p53 target genes. The PXXP domain (proline-rich domain) plays an important role in the apoptotic activity of p53. The DNABD domain (DNA-binding domain, DNA-binding domain, “zinc finger”) directly interacts with DNA. NLS (nuclear localization domain) is a nuclear localization domain, OD (oligomerization domain) is an oligomerization (tetramerization) domain, NEG (negative-regulation domain) is a negative regulation domain that ensures the detachment of DNABD from DNA

The p53 protein is encoded by the *TP53* gene, located on chromosome 17p13.1. It comprises 11 exons, with the first being non-coding. There are at least 12 isoforms of the p53 protein: p53 (or p53α), p53 (β, γ), Δ40p53 (α, β, γ), Δ133p53 (α, β, γ), and Δ160p53 (α, β, γ) (Fig. 1). Various protein isoforms arise from molecular mechanism variations: alternative splicing (intron 2, α/β/γ segments), alternative promoters (P1 and P2), alternative translation initiation (start codons 1, 40, 133, 160) [6]. These p53 isoforms exhibit distinct functional features, and their cellular proportions vary depending on the type of healthy or tumorous tissue. The canonical form of p53 is most common in the cell. Among all isoforms, it possesses the largest molecular mass (53 kDa), consisting of 393 amino acids, and is divided into 7 domains (Fig. 1).

**Fig. 2:**
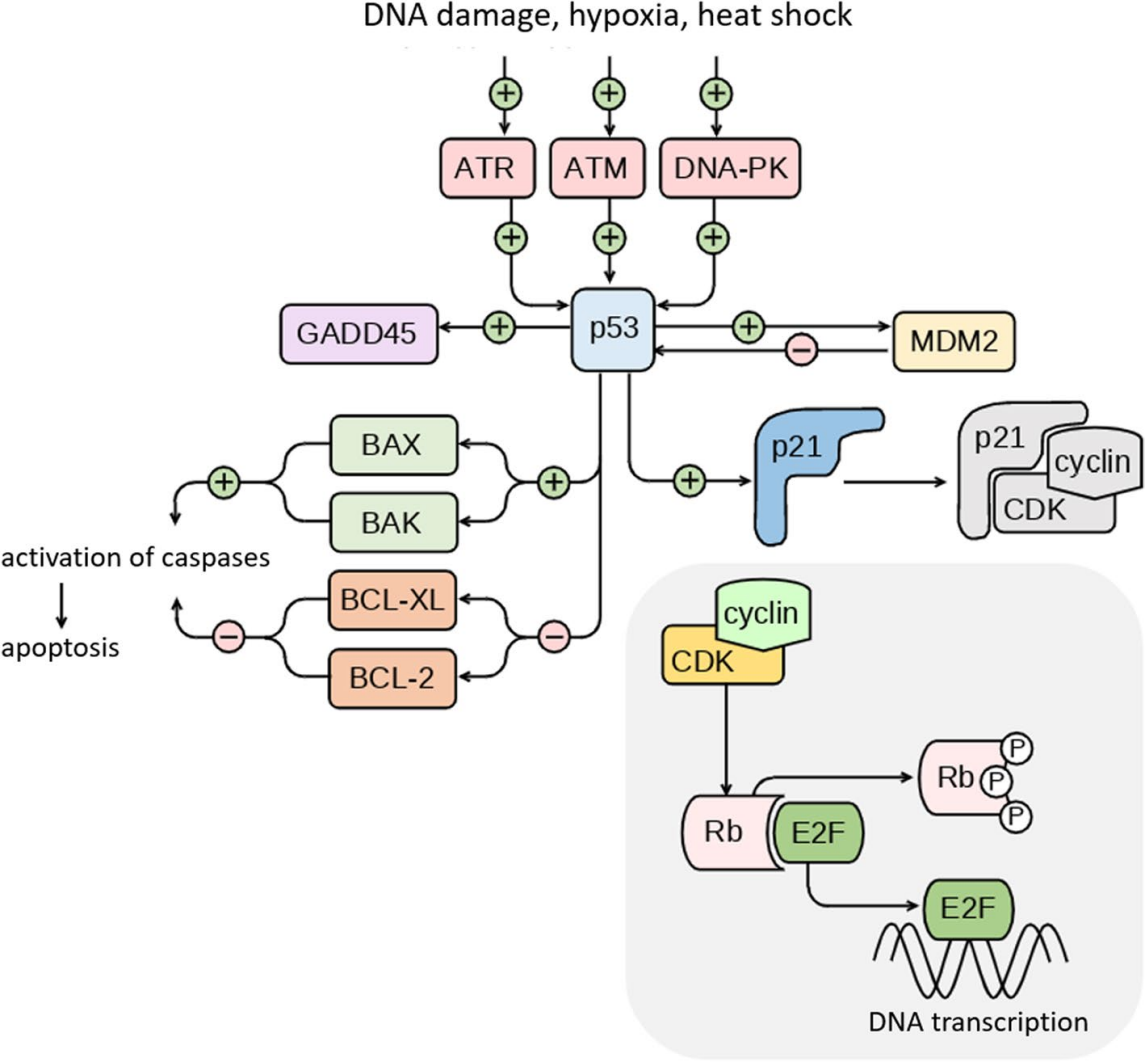
The main signaling pathways of the p53 protein in the cell. DNA damage caused by various stress factors (UV radiation, radiation, viral infection) activates protein kinases ATR, ATM, DNA-PK, which belong to the PIKK family. One of the signaling pathways of these protein kinases includes the p53 protein, the degradation of which as a result slows down and its concentration increases. The p53 protein acts as a transcription factor, activating some genes (GADD45, MDM2, BAX, BAK, p21) and suppressing others (BCL-2, BCL-XL). In some signaling pathways, p53 interacts with other proteins, for example with mitochondrial anti-apoptotic proteins during the initiation of apoptosis [6]. One of the protein regulators of p53 is MDM2, which interacts with p53 according to the principle of negative feedback. The p53 protein enhances MDM2 transcription, while MDM2 acts as a ubiquitin ligase to promote p53 degradation. The p21 protein inhibits the complex of cyclin and cyclin-dependent kinases, in the event of DNA damage, preventing the transition from G1 to S phase and thus stopping the cell cycle. In the absence of the p21 protein, the cyclin-dependent kinase CDK phosphorylates the Rb protein, and the transcription factor E2F is involved in DNA synthesis. If DNA damage cannot be repaired, p53 initiates apoptosis, including regulating the expression of bcl-2 family genes

The p53 protein is activated in response to DNA damage, hypoxia, metabolic dysfunction, heat shock, and oncogene expression (Fig. 2). It ensures genome stability by performing various functions, including cell cycle control, initiation of DNA damage re-pair, and, in cases where restoration of the defect is impossible, triggering apoptosis. In the absence of stress stimuli, the concentration of p53 in the cell remains low and is maintained through a balance between its synthesis and degradation. Excess amount of p53 can lead to programmed cell death, while a deficiency of one increases the malignant transformation risk. By impeding the proliferation of cells with damaged DNA, p53 serves as a crucial oncosuppressor [7].

Mutations in the *TP53* gene can affect the function of the p53 protein in different ways:

1. Loss-of-function (LOF): Complete or partial loss of the wild-type protein function.
2. Dominant-negative (DN): Dominant-negative effect, where the mutant protein forms tetramers with the wild-type protein and suppresses p53 function in the cell.
3. Gain-of-function (GOF): Acquisition of atypical functions, such as the ability to activate promoters atypical for the p53 protein.

Various mechanisms can implement each of these effects, and they may coexist.

Testing for *TP53* gene mutation carriage should be conducted before initiating treatment, as the test result can influence the therapeutic strategy. Recommendations for *TP53* gene mutation testing are presented in Table 1 [8]. Having an increased risk of developing MN at other sites, patients carrying *TP53* mutations should be recommended to avoid radiotherapy and DNA-toxic chemotherapy. In these cases, surgical methods should be preferred when choosing a treatment strategy [9].

**Table 1:** Recommendations for *TP53* gene germline variants testing (2020) [10]

Recommendation 1	Patients meeting the modified Chompret criteria: *— Familial presentation*: proband with a *TP53* core tumor (breast cancer, soft-tissue sarcoma, osteosarcoma, central nervous system tumor, adrenocortical carcinoma) before 46 y.o. AND at least one first- or second-degree relative with a core tumor before 56 y.o. or *- Multiple primitive tumors*: proband with multiple tumors, including 2 *TP53* core tumors, the first of which occurred before 46 y.o., irrespective of family history; or—*Rare tumors*: patient with adrenocortical carcinoma, choroid plexus carcinoma, or rhabdomyosarcoma of embryonal anaplastic subtype, irrespective of family history; or *- Very early-onset breast cancer:* Breast cancer before 31 y.o., irrespective of family history
Recommendation 2	Children and adolescents should be tested for germline *TP53* variants if presenting with: • Hypodiploid acute lymphoblastic leukemia (ALL); or • Otherwise unexplained sonic hedgehog-driven medulloblastoma; or • Jaw osteosarcoma
Recommendation 3	Patients who develop a second primary tumor, within the radiotherapy field of a first core *TP53* tumor which occurred before 46 y.o., should be tested for germline *TP53* variants
Recommendation 4	a. Patients older than 46 y.o. presenting with breast cancer without personal or familial history fulfilling the Chompret Criteria should not be tested for germline *TP53* variants b. Any patient presenting with isolated breast cancer and not fulfilling the Chompret Criteria, in whom a disease-causing *TP53* variant has been identified, should be referred to an expert multidisciplinary team for discussion
Recommendation 5	Children with any cancer from southern and south-eastern Brazilian families should be tested for the p.R337H Brazilian founder germline *TP53* variant

Only isolated cases of Russian families with LFS diagnosis, are published elsewhere. The exact incidence of Li-Fraumeni syndrome among Russian cancer patients has not yet been defined. The aim of this study was to investigate the frequency and spectrum of *TP53* pathogenic variants associated with LFS in a large cohort of mainly of breast cancer patients from Russia. Also, the genotype–phenotype correlations from 13 unrelated patients diagnosed with *TP53*-associated MN are presented.

## Methods

This study analyzed the mutational distribution of clinically significant *TP53* germline variants in 3455 patients with diagnosed cancer and suspected hereditary cancer syndrome. These patients were identified across various medical institutions using molecular genetic methods based on Next-Generation Sequencing (NGS): multi-gene NGS panel testing (1655 studies) and whole-genome sequencing (WGS, 1800 studies).

Out of the total cohort, 3247 were females (94%) and 208 were males (4%). Among the examined individuals, 2957 were diagnosed with breast cancer (85.6%), including cases occurring before the age of 31 (100/2957, 3.4%), bilateral breast cancer (138/2957, 4.7%), and breast cancer as part of MPMN involving other locations (205/2957, 6.9%). Addition-ally, 498 individuals (14.4%) presented with tumors in the ovaries, colon, pancreas, and other locations. The average age of solid tumor manifestation in the studied group of 3455 patients was 46.47 y.o. (95% CI: 46.12–46.83; range 11–86 y.o.y.o.).

NGS Panels. The study involved analyzing 1655 peripheral blood samples from patients receiving specialized care at the Moscow Clinical Research Center named after A.S. Loginov. This analysis utilized multi-gene custom NGS panels in the laboratories of the Centre for Strategic Planning and Management of Biomedical Health Risks (Moscow, Russia), Engelhardt Institute of Molecular Biology of Russian Academy of Sciences (Moscow, Russia) and Kazan Federal (Kazan, Russia) using the Illumina MiSeq sequencer (Tables 2 and 3). Sample preparation, sequencing, and bioinformatic processing followed the methodology previously described in article [11].

**Table 2:** Gene list for the NGS-panels

Laboratory	Number of samples	Number of studied genes	Genes
Kazan Federal University	166	61	*APC, ATM, ATR, BARD1, BLM, BRCA1, BRCA2, BRIP1, BUB1, CDH1, CDKN2A, CHEK1, CHEK2, CTNNA1, EPCAM, ERCC1, ERCC2, FAM175A, FANCB, FANCC, FANCD2, FANCF, FANCG, FANCI, FANCL, FANCM, MCPH1, MDM1, MLH1, MRE11A, MSH2, MSH3, MSH6, MUTYH, NBEAL1, NBN, NF1, PALB2, PMS2, POLD1, POLE, PPM1D, PTEN, RAD50, RAD51C, RAD51D, RAD52, RAD54B, RBBP8, RECQL4, RINT1, SETBP1, SLX4, SMAD4, STK11, ****TP53****, TP53BP1, TSC1, TSC2, XPC, XRCC2* including promoter regions
Engelhardt Institute of Molecular Biology	833	60	*ATM, BRCA1, PALB2, RAD50, MRE11A, NBN, RAD51D, RAD51C, RAD54B, BLM, XRCC2, TP53, BRCA2, BARD1, ATR, CHEK2, FANCM, RECQL4, FANCF, FANCI, FANCC, FANCG, FANCL, MLH1, CDKN2A, MSH2, MSH6, PTEN, CHEK1, BRIP1, RBBP8, SLX4, FAM175a, RAD52, RINT1, CDK12, CDH1, STK11, PMS2, MUTYH, PPM1D, APC, MCPH1, NF1, EPCAM, BUB1, FANCD2, ****TP53****, BP1, MRE11, POLD1, POLE, SMAD4, MSH3, CTNNA1, ERCC2, FANCB, ERCC1, TSC1, TSC2, XPC*
Centre for Strategic Planning and Management of Biomedical Health Risks	656	44	*APC, ATM, AXIN2, BARD1, BLM, BMPR1A, BRCA1, BRCA2, BRIP1, CDH1, CDKN2A, CHEK2, DICER1, EPCAM, GALNT12, GREM1, MEN1, MLH1, MLH3, MSH2, MSH3, MSH6, MUTYH, NBN, NF1, NTHL1, PALB2, PMS2, POLD1, POLE, PTCH1, PTCH2, PTEN, RAD51C, RAD51D, RET, SMAD4, STK11, SUFU, ****TP53****, TSC1, TSC2, VHL, WT1*

**Table 3:** Samples for NGS-testing

Name of the medical organization that carried out the collection of biosamples	Research method	Number of samples	Executing Lab
Moscow Clinical Scientific Center Named after Loginov	NGS panel	166	Kazan Federal University
	NGS panel	656	Centre for Strategic Planning and Management of Biomedical Health Risks
	NGS panel	833	Engelhardt Institute of Molecular Biology
	WGS	716	LLC Evogen
City Clinical Oncological Hospital No. 1	WGS	768	LLC Evogen
Moscow City Clinical Hospital named after S.P. Botkin	WGS	88	LLC Evogen
Moscow City Clinical Hospital No.62	WGS	112	LLC Evogen
Moscow City Clinical Hospital named after D.D. Pletnev	WGS	40	LLC Evogen
Moscow Medical Cluster "Kommunarka"	WGS	76	LLC Evogen
**Total**	**3455**		

WGS was conducted on 1800 patients receiving treatment in six state-funded healthcare institutions in Moscow: Moscow City Clinical Hospital No.1, Moscow Clinical Scientific Center Named after Loginov, Moscow City Clinical Hospital No.62, Moscow City Clinical Hospital named after S.P. Botkin, Moscow City Clinical Hospital named after D.D. Pletnev, and Moscow Medical Cluster "Kommunarka". The patient selection criteria, materials, and methods are detailed in article [12].

The methodology of molecular genetic diagnostics, quality control, and genome-wide sequencing, as well as the examination of gene panels, was described in our previous article [13, 14].

The clinical significance of nucleotide sequence variants was analyzed according to the recommendations of the American College of Medical Genetics and Genomics (ACMG). This analysis employed specialized bioinformatic algorithms and databases, including OMIM (Online Mendelian Inheritance in Man), NCBI (National Center for Bio-technology Information), VarSome (The Human Genomics Community), and others, as well as scientific literature data. Population frequencies of identified variants were assessed using the gnomAD (Genome Aggregation Database).

This study presents the carrier status results of clinically significant variants in the coding region of the *TP53*, 20 base pairs proximal to the 5' end and 20 base pairs distal to the 3' end of each exon have been analyzed. Point mutation, microinsertion, deletion and duplication (< 20 bp) at exon level can be simultaneously detected. Certain copy number variants (CNV) such as large fragment of heterozygous gene, special mutations such as dynamic mutation, complex recombination, structural variants (e.g.: large fragment deletion, duplication and inversion rearrangement), large fragment heterozygous insertion (e.g.: Alu-induced insertion) as well as mutations in gene regulatory region and deep intronic region was excluded from calculations to facilitate a valid comparison of results between NGS panels and WGS.

Clinically significant variants were validated using the Sanger sequencing. Data processing was performed using GraphPad Prism v.9.5.1 (https://www.graphpad.com/) through descriptive statistical methods: proportions are presented in percentages, means with 95% confidence intervals, and statistical tests considered a significance level of *p* < 0.05.

## Results

In a comprehensive analysis of 3455 genomic DNA samples from breast cancer patients, 13 individuals (0.4%) unrelated to each other were identified with clinically significant germline variants in the *TP53* gene. Notably, all mutation carriers were females, with either unilateral breast cancer (9/13; 69.2%) or breast cancer as part of MPMN (4/13; 30.8%): bilateral breast cancer in 3 cases and Hodgkin lymphoma in 1 case. The average age of manifestation of breast cancer was 39.9 y.o. (95% CI: 33.1–46.8, range 28–66 y.o.), with a median age of manifestation – 36.0 y.o.

**Fig. 3:**
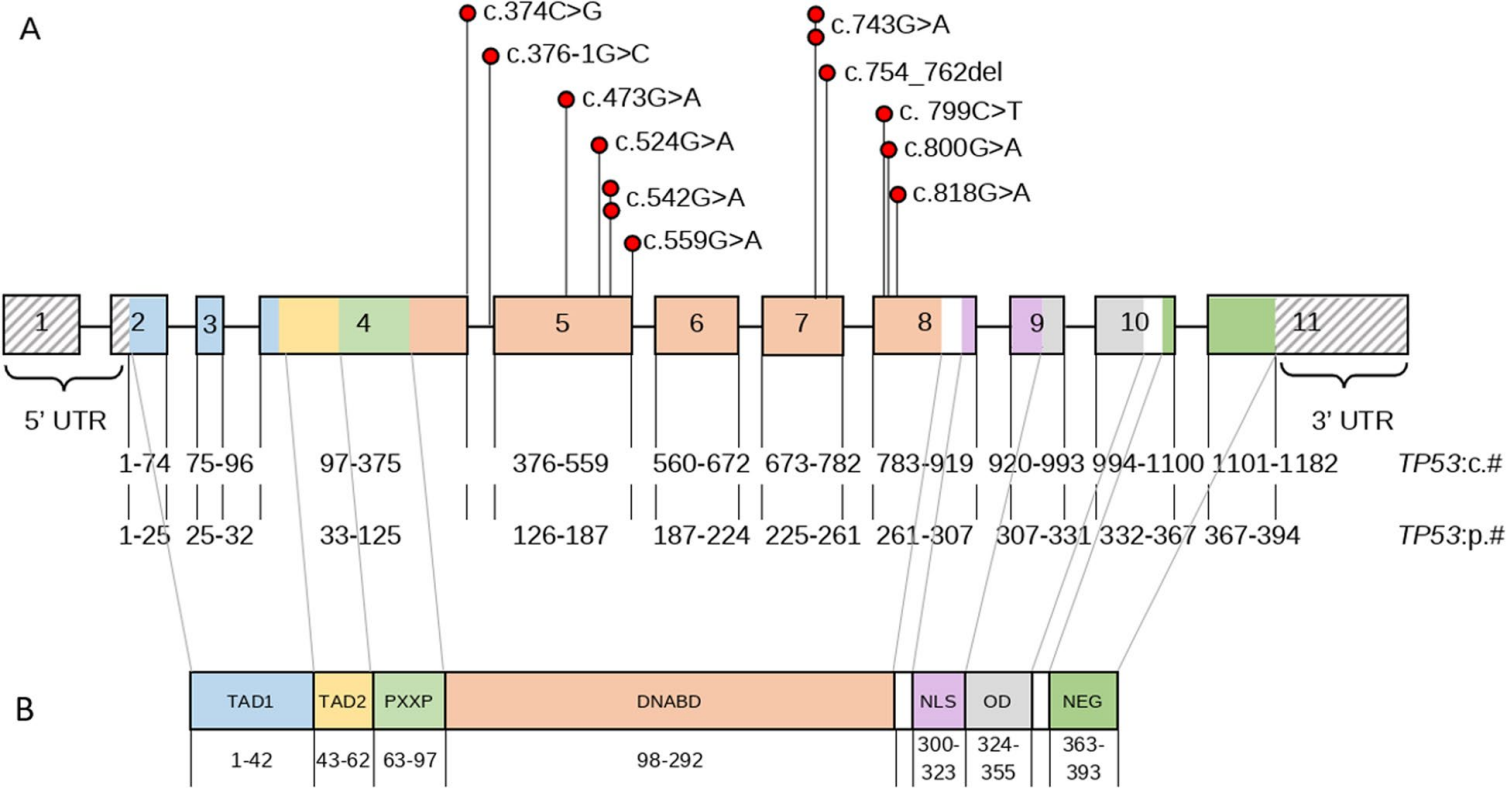
Structure and mutations of the *TP53* gene (NM_000546.6). A. Spectrum of identified mutations in the *TP53* gene. B. Domains of protein p53 [15]

Among the *TP53* mutation carriers, 84.6% (11/13) had pathogenic variants (P), 15.4% (2/13) had likely pathogenic variants (LP). In 11 cases missense variants in 1 case 9 bp exon 7 in frame deletion (c.754_762del) and in 1 case splice acceptor variant in intron 4 (c.376-1G > C) were identified. All detected variants affected regions of the *TP53* gene associated with the synthesis of the DNA-binding domain (DNABD) of the p53 protein (Fig. 3).

Among the surveyed patients, 38.5% (5/13) met the modified Chompret criteria. The remaining 61.5% (*n* = 8) of individuals diagnosed with breast cancer were older than 31 y.o. at diagnosis, and their family history did not align with recommendations for *TP53* mutation testing. Clinical and family history characteristics of patients with identified variants are presented in Table 4.

**Table 4:** Clinical and anamnestic characteristics of patients with identified *TP53* variants

	**Sex**	**MN sites**	**Age (diagnosis)**	**Criteria Chompret**	**Family history**	**SNV (hg38) NM_000546.5**	**SNV location in the *TP53* gene**	**ACMG classification**	**LOF class, func**	**SpliceAI results (delta scores)**
P01	F	MPMN: 1) right breast	36	No	Mother—breast cancer, died at 28	chr17:g.7675995G > C c.374C > G p.Thr125Arg	Ex 4	P	LOF + , NF	AL 0.07 DL 0.07 AG 0.00 DG 0.15
		2) left breast	38							
P02	F	left breast	36	Yes	CNS Sarcoma	chr17:g.7675237C > G c.376-1G > C	Int 4	P	N/A, N/A	AL 1.00 DL 0.04 AG 0.98 DG 0.04
P03	F	right breast	35	Yes	CNS Stomach Sarcoma	chr17:g.7675139C > T c.473G > A p.Arg158His	Ex 5	P	LOF + , NF	AL 0.00 DL 0.00 AG 0.00 DG 0.00
P04	F	left breast	28	Yes	negative	chr17:g.7675088C > T c.524G > A p.Arg175His	Ex 5	P	LOF + , NF	AL 0.00 DL 0.00 AG 0.00 DG 0.01
P05	F	right breast	38	No	breast cancer Lungs	chr17:g.7675070C > T, c.542G > A, p.Arg181His	Ex 5	P	LOF-, PF	AL 0.01 DL 0.00 AG 0.00 DG 0.02
P06	F	MPMN: 1) right breast	47	No	breast cancer Lungs	chr17:g.7675070C > T, c.542G > A, p.Arg181His	Ex 5	P	LOF-, PF	AL 0.01 DL 0.00 AG 0.00 DG 0.02
		2) left breast								
P07	F	left breast	33	No	negative	chr17:g.7675053C > T, c.559G > A, p.Gly187Ser	Ex 5	LP	LOF-, N/A	AL 0.12 DL 0.05 AG 0.00 DG 0.56
P08	F	MPMN: 1) Hodgkin's lymphoma IIIBst (variant of nodular sclerosis)	20	Yes	Maternal aunt—breast cancer at 50 y.o	chr17:g.7674220C > T, c.743G > A, p.Arg248Gln	Ex 7	P	LOF + , NF	AL 0.00 DL 0.00 AG 0.00 DG 0.00
		2) left breast	29							
P09	F	right breast	49	No	Mother—breast cancer at 55 y.o	chr17:g.7674220C > T, c.743G > A, p.Arg248Gln	Ex 7	P	LOF + , NF	AL 0.00 DL 0.00 AG 0.00 DG 0.00
P10	F	left breast	29	Yes	negative	chr17:g.7674200 TGATGGTGAG > T c.754_762del, p.Leu252_Ile254del	Ex 7	P	N/A, N/A	AL 0.01 DL 0.00 AG 0.03 DG 0.00
P11	F	MPMN: 1) right breast	55	No	Grandmother—CC at 75 y.o	chr17:g.7673821G > A c. 799C > T p.Arg267Trp	Ex 8	P	LOF-, N/A	AL 0.02 DL 0.00 AG 0.04 DG 0.00
		2) left breast								
P12	F	right breast	38	No	negative	chr17:7673820C > T c.800G > A p.Arg267Gln	Ex 8	LP	LOF-, PF	AL 0.04 DL 0.00 AG 0.08 DG 0.00
P13	F	left breast	66	No	Maternal grandmother—RC	chr17:7673802C > T, c.818G > A, p.Arg273His	Ex 8	P	LOF + , NF	AL 0.02 DL 0.00 AG 0.01 DG 0.00

The Cancer Pedigrees, see Fig. 3. The SpliceAI results column contains delta scores acquired from SpliceAI in silico predictor [16]. Delta scores range from 0 to 1 and can be interpreted as the probability that the variant affects splicing at any position within a window around it (in our case—± 500 base pairs window was used). All the delta scores exceeding the recommended cutoff (0.5) were highlighted in red

*Abbreviations MN* Malignant neoplasms, *BC* breast cancer, *CC* colon cancer, *RC* rectal cancer, *MPMN* multiple primary malignant neoplasms, *ACMG* recommendations American College of Medical Genetics and Genomics on the interpretation of genetic variants, *P* pathogenic, *LP* likely pathogenic, *LOF* ± – “loss of function” presence [17], *NF* non-functional variant, *PF* partially functional [18], *N/A* no data available, *AL* acceptor loss score, *DL* donor loss score, *AG* acceptor gain score, *DG* donor gain score

**Fig. 4:**
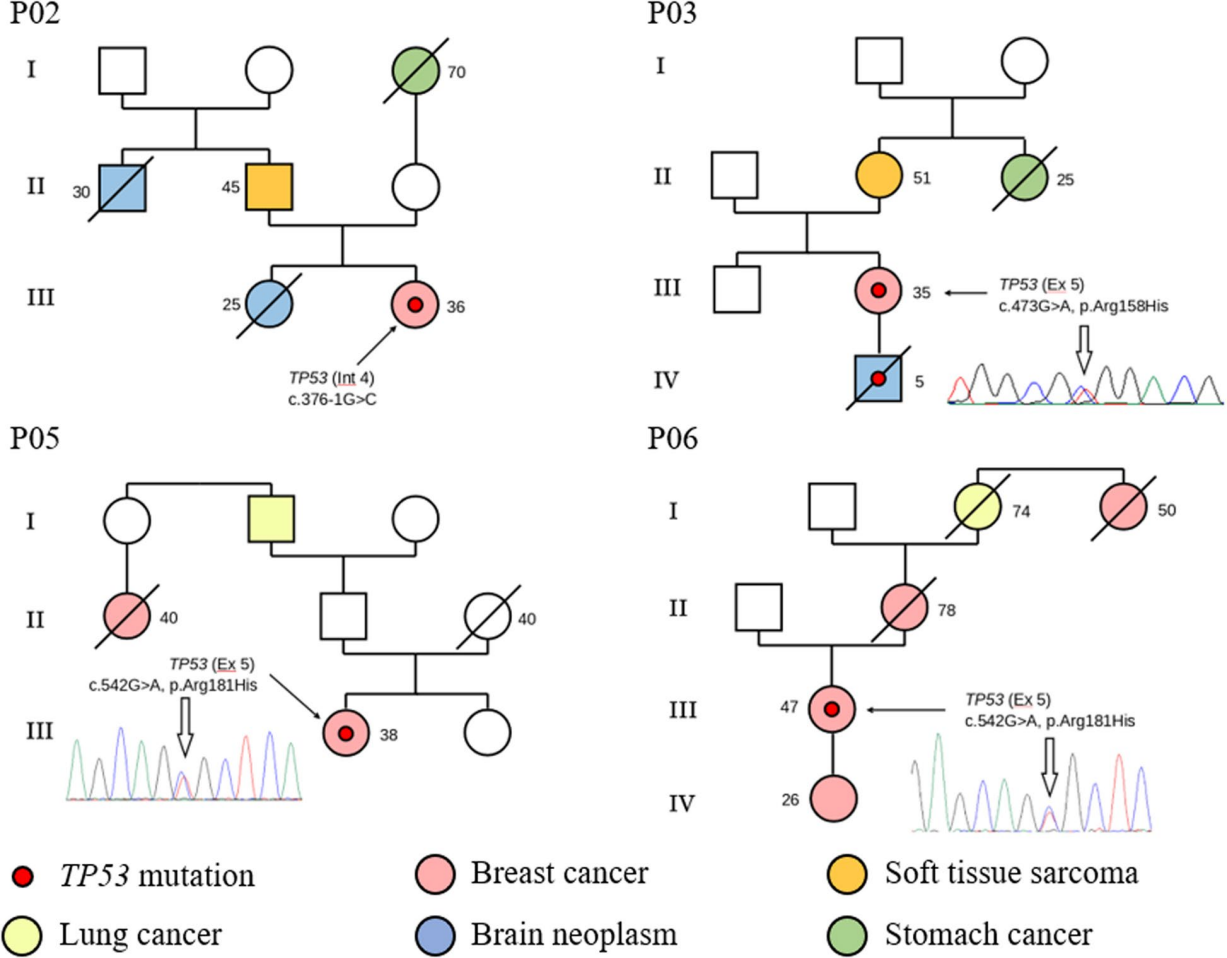
Pedigrees of patients with cancer among relatives in several generations (P02, P03, P05, P06). The onset age for alive relatives and the death age if a relative died. Our study also revealed two pairs of unrelated patients with recurring *TP53* variants (c.542G > A and c.743G > A)

A notable 69.2% (9/13) of patients reported a family cancer history. Among them, 38.4% (5/13) had first or second-degree relatives with confirmed breast cancer. In 30.8% (4/13) of cases, there was a clustering of oncological diseases across multiple generations, illustrated in the pedigrees of these patients in Fig. 4.

Histological types in breast cancer included unspecified invasive carcinoma (13/15, 86.6%), ductal carcinoma in situ with foci of invasion (1/15, 6.6%), and ductal carcinoma in situ (1/15, 6.6%). The clinical and morphological features of breast cancer in *TP53* mutation carriers are outlined in Table 5.

**Table 5:** Clinical and morphological features of breast cancer in patients with mutations in the *TP53* gene

	**Sex**	**MN sites**	**Age (diagnosis)**	**Stage**	**HT**	**Grade**	**ER, % or score**	**PR, % or score**	**Ki67, %**	**Her2**	**office**
P01	F	MPMN: 1) right breast	36	cT2N1M1, mts to the lungs, IV st	IUC	N/A	80–90%	80%	70	HER2 + (2 + , FISH +)	Lum B Her2 +
		2) left breast	38	cT2N3Mx	IUC	G3	0	0	90	HER2-	Triple-
P02	F	left breast	36	pT1cN0M0, IA st	IUC	G2	4	6	35	HER2 +	Lum B Her2 +
P03	F	right breast	35	pT1N0M0, IA st	IUC	G2	5	4	52	HER2-	Lum B Her2-
P04	F	left breast	28	cT2N0M0, IIa st	IUC	G3	0	0	70	HER2-	Triple-
P05	F	right breast	38	pT2N2M0, IIIA st	N/A	N/A	pos	pos	N/A	HER2-	Lum B Her2-
P06	F	MPMN: 1) right breast	47	pT1N0M0, IA st	IUC	G2	90%, 8	99%,8	23	HER2-	Lum B Her2-
		2) left breast		pTisN0M0 0 st	DC in situ	–-	–-	–-	–-	–-	–-
P07	F	left breast	33	cT2N0M0, IIA st	IUC	G2	8	8	50	HER2-(1 +)	Lum B Her2-
P08	F	MPMN: 1) Hodgkin's lymphoma IIIB	20	–-	–-	–-	–-	–-	–-	–-	–-
		2) left breast	29	T3N3M0, IIIC Art	IUC	G2	0	0	90	HER2-	Triple-
P09	F	right breast	49	pT2N1M0, IIB st	DC in situ with foci of invasion	N/A	0	0	30	HER2 + (3 +)	HR- Her2 +
P10	F	left breast	29	pT2N0M0, IIA st	IUC	G3	8	5	40	HER2 + (3 +)	Lum B Her2 +
P11	F	MPMN: 1) right breast	55	st4bN1fM0, IIIB st	IUC	G3	8	6	50	HER2-(1 +)	Lum B Her2-
		2) left breast		cT1bN0M0, IA st	IUC	G2	8	6	30	HER2 + (3 +)	Lum B Her2 +
P12	F	right breast	38	ycT4bN2fM0, IIIB st	IUC	G3	0	0	32	HER2 + (3 +)	HR- Her2 +
P13	F	left breast	66	cT2N0M0, IIA st	IUC	G2	0	0	N/A	HER2-	Triple-

*Abbreviations*: *HT* histological type, *ER* estrogen receptors, *PR* progesterone receptors, *pos*. positive receptor status, *MBT* molecular biological subtype, *IUC* invasive unspecified carcinoma, *DC* in situ – ductal carcinoma in situ, Lum – luminal subtype, *HR*- non-luminal subtype, *Triple-* triple negative subtype, *N/A* no data available

## Discussion

The frequency of identified *TP53* clinically significant germline variants was 0.4% (13/3455) and in all cases *TP53* mutations were identified in females with breast cancer. Therefore, the proportion of *TP53*-associated breast cancer within all breast cancer cases in our cohort was 0.44% (13/2957), consistent with existing literature [19]. The predominance of this localization of cancer types is attributed to the high prevalence of breast cancer patients in our cohort and the elevated risk of breast cancer in the spectrum of tumors typical for LFS.

Among patients with breast cancer aged up to 31 y.o., the proportion of carriers of pathogenic *TP53* variants was 3.0% (3/100), aligning with previously reported rates of 3.8%-6.0% for patients ≤ 31 y.o. [19, 20].

According to the *TP53* Database (version R20, ISB-CGC, https://Tp53.isb-cgc.org/), the most frequent germline variants in the *TP53* gene are located at positions Arg175, Arg213, Gly245, Arg248, Arg273, Arg282, and Arg337, accounting for 40% (937/2358) of missense mutations in the database. Excluding the position Arg337, characteristic of the southern Brazilian population, these positions account for 32% of mutations. In our study, variants categorized as frequent according to the *TP53* Database accounted for 30.8% of the identified *TP53* variants: c.524G > A (Arg175; *n* = 1; 7.7%), c.743G > A (Arg248; *n* = 2; 15.4%), and c.818G > A (Arg273; *n* = 1; 7.7%). The majority of *TP53* variants in our study, consistent with scientific literature, were represented by missense variants within the DNABD domain.

One *TP53* variant identified in our study absent in the GnomAD population frequency database and are not described in literature or databases. In patient P02, harboring the variant c.376-1G > C in intron 4, the family history and clinical features align with Chompret criteria (Fig. 4). Scientific literature describes a different nucleotide substitution in the same DNA position—c.376-1G > A, clinically significant, disrupting the canonical splice acceptor site and detected in patients exhibiting features of LFS [21–24]. Such genetic alterations predominantly result in protein loss of function (LOF) [25]. Based on this data, the previously undescribed variant is annotated as pathogenic.

In the present study, it was observed that a significant proportion of *TP53* pathogenic variants appear to occur outside of LFS families, which is a noteworthy finding. However, a recent report from a larger study indicates a higher proportion of carriers complying with the modified Chompret criteria [26]. This discrepancy may be attributed to the lower sensitivity of the Chompret criteria in our study, which could be due to the small number of patients in the group of *TP53* mutation carriers. A larger cohort may provide a more comprehensive understanding of the relationship between *TP53* variants and compliance with the Chompret criteria, highlighting the need for further investigation in this area.

Our study revealed two pairs of unrelated patients harboring described pathogenic variants. The first pair (P05 and P06) exhibited variant c.542G > A in exon 5, leading to the p.Arg181His substitution. The minor allele frequency is 0.00001314 (GnomAD Genomes). Patient P05, aged 38, was diagnosed with unilateral breast cancer, whereas patient P06, aged 47, was diagnosed with synchronous bilateral breast cancer. Both reported familial history of breast cancer and lung cancer (Fig. 4).

The second pair (P08 and P09) exhibited variant c.743G > A in exon 7, resulting in the p.Arg248Gln substitution. The substitution at position Arg248 is the most prevalent according to *TP53* Database, accounting for 9.1% of all described germline pathogenic *TP53* variants. Patient P09, aged 49, had unilateral breast cancer, while patient P08 had Hodgkin's lymphoma at age 20 and breast cancer at age 29. Both reported a familial history of breast cancer (Table 4).

Our study involved 138 patients with synchronous/metachronous bilateral breast cancer, accounting for 4.7% (138/2957) of all breast cancer patients. Among *TP53* mutation carriers, 23.1% (3/13) exhibited bilateral breast cancer (OR = 5.9289, 95% CI: 1.6130–21.7933, *p*-value = 0.0074). Kwong A. et al. [27] results indicated an OR = 7.0011 (95% CI: 2.8449–17.2292, *p*-value < 0.0001), aligning with our findings. Thus, synchronous/metachronous bilateral breast cancer is significantly more prevalent among breast cancer patients with *TP53* gene mutations compared to those without mutations.

### Functional characteristics of TP53 mutations

**Fig. 5:**
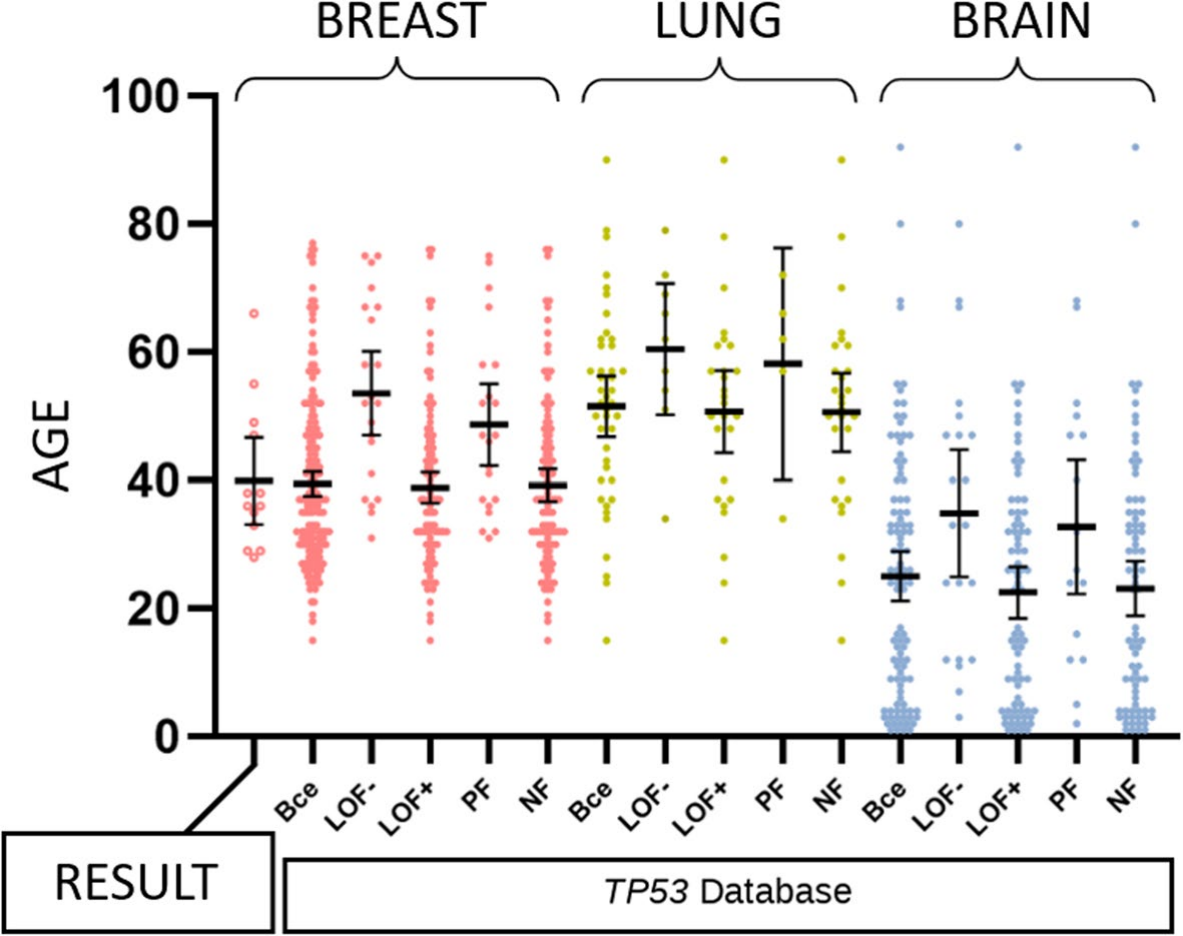
Age of manifestation of cancer depending on the location and functional type of mutation. LOF ± – presence or absence of the loss of function effect according to Giacomelli et al. [17], NF is a non-functional variant, PF is a partially functional variant according to data from [18]. The majority of mutations in the *TP53* gene identified in our study are classified as LOF + /NF. LOF—/PF mutations were detected in 5 patients (P05, P06, P07, P11, P12). Given the wide age range at manifestation for each functional mutation type, this parameter does not allow to perform precise prognosis for a specific carrier. However, it may be valuable for assessing the clinical significance of the variant and evaluating the risk of early manifestation of cancer in a family

In the study by Giacomelli AO et al. [17] an experimental assessment of various *TP53* gene mutations impact on p53 protein function was demonstrated. Several thousands malignant tumor cell lines with different *TP53* mutations were analyzed, subjected to substances activating p53: nutlin-3 (inhibitor of MDM2 and p53 binding) or etoposide (topoisomerase II inhibitor, causing DNA damage). The presence of Dominant Negative (DN) and Loss of Function (LOF) effects for each mutation was determined through experiments. Analyzing *TP53* Database data reveals that these characteristics statistically significantly influence the age of disease manifestation (Fig. 5). For breast cancer patients the average age of manifestation is 38.9 y.o. with LOF + (95% CI: 36.4–41.3) and 53.6 y.o. (95% CI: 47.0–60.1) with LOF— mutations. This pattern holds true for malignant tumors at other locations. For lung cancer, the average age of manifestation is 50.7 (95% CI: 44.3–57.1) and 60.4 (95% CI: 52.2–70.7) y.o., and for brain cancer, it is 22.5 (95% CI: 18.5–26.6) and 34.9 (95% CI: 25.0–44.8) y.o. (LOF + and LOF- respectively).

In the study by Kato S et al. [18] the assessment of p53 protein function with different mutations was conducted using a different method. For each *TP53* mutation in yeast culture the median transcriptional activity of p53 was calculated across 8 specific promoters (activity expressed as a percentage of wild-type protein activity). Missense variants were classified as "non-functional" if the median was ≤ 20%; "partially functional" if the median was > 20% and ≤ 75%; "functional" if the median was > 75% and ≤ 140%; and finally, "super-functional" if the median was > 140. According to scientific literature in *TP53* Database most breast cancer patients with breast cancer are carriers of non-functional (107/133) or partially functional (21/133) variants, with an average age of tumor manifestation of 39.2 (95% CI: 36.7–41.8) and 48.7 (95% CI: 42.3–55.1) y.o. respectively. Functional and super-functional variants (5/133) in breast cancer patients are classified as benign or likely benign according to ACMG criteria.

Thus, functional features of the p53 protein determined in cell culture experiments can help determine the clinical significance of different *TP53* mutations.

## Conclusion

Our study presents a spectrum of pathogenic and likely pathogenic germline variants of the *TP53* gene in a large cohort of Russian patients diagnosed with various cancers, along with clinical characteristics and family oncological history of carrier patients. All clinically significant *TP53* gene variants were identified in women with breast cancer (including bilateral breast cancer and breast cancer as part of multiple primary malignant neoplasms). Only 5 out of 13 mutation carriers (38.5%) met modified Chompret criteria, indicating candidates for molecular-genetic testing of the *TP53* gene. Therefore, relying solely on these criteria in clinical practice for determining indications for genetic testing would fail to identify a half of the mutation’s carriers. This conclusion should be considered in medical-genetic counseling and molecular-genetic searches in cases of suspected hereditary cancer, particularly LFS syndrome in patients diagnosed with breast cancer.

## Data Availability

No datasets were generated or analysed during the current study.
